# Short-Term Relief or Long-Term Repair: A Narrative Review of Corticosteroid and Platelet-Rich Plasma Injections in Rotator Cuff Tendinopathy

**DOI:** 10.7759/cureus.97271

**Published:** 2025-11-19

**Authors:** Olive Kyaw, Chan Khin

**Affiliations:** 1 Trauma and Orthopaedics, University Hospital Sussex NHS Foundation Trust, Brighton, GBR

**Keywords:** corticosteroid injection, platelet-rich plasma, randomised controlled trial, rotator cuff tendinopathy, shoulder pain, systematic review, tendon healing

## Abstract

Rotator cuff tendinopathy is a common cause of shoulder pain and disability in adults over 40. Corticosteroid injections provide short-term pain relief but may impair tendon healing with repeated use. Platelet-rich plasma (PRP) aims to promote tissue repair; however, comparative evidence remains heterogeneous. We conducted a narrative review of comparative studies of corticosteroid versus PRP injections for rotator cuff tendinopathy. Ovid MEDLINE, Embase, and PubMed were searched for English-language studies (2010-25). Eligible designs included randomised controlled trials (RCTs), prospective comparative studies, and systematic reviews/meta-analyses. Of 263 records, 60 full texts were screened, and 17 studies were included. Reviews/meta-analyses consistently found corticosteroids superior for short-term relief (<3 months), with several suggesting PRP may be favoured for mid- to long-term outcomes (6-12 months), though results were inconsistent. Among RCTs, early evidence showed no benefit of PRP over placebo, whereas more recent trials reported improved pain and function with PRP versus corticosteroids; other RCTs found no difference. Prospective studies generally suggested more sustained outcomes with PRP. Heterogeneity in PRP preparation and protocols limits comparability. Corticosteroids remain useful for rapid symptom control, but benefits wane and repeated use may pose risks. PRP may offer more durable improvements for selected patients, albeit with variable effect sizes across studies and higher cost.

## Introduction and background

Rotator cuff tendinopathy is one of the most common causes of shoulder pain, affecting both athletic and general populations [1,2]. It accounts for a significant proportion of musculoskeletal consultations, particularly in individuals over the age of 40 [3,4]. The condition leads to pain, reduced function, and impaired quality of life, and in chronic cases may progress to partial or full-thickness rotator cuff tears [5,6].

Conservative management, including physiotherapy, non-steroidal anti-inflammatory drugs, and activity modification, is the first line of treatment. When symptoms persist, injection therapies are commonly used as adjuncts. Corticosteroid injections have been widely utilised for decades due to their potent anti-inflammatory properties, and they provide short-term pain relief [7,8]. However, concerns exist regarding potential tendon degeneration, risk of recurrence, and diminishing effectiveness with repeated use [9].

Platelet-rich plasma (PRP) has emerged as a biologic alternative aimed at stimulating tendon healing. PRP delivers growth factors and cytokines that may promote tissue healing, with evidence suggesting longer-lasting benefits compared with corticosteroids [10-12]. Despite growing interest, heterogeneity in PRP preparation methods, injection protocols, and study populations has led to conflicting findings across trials and systematic reviews [13,14].

Given the high prevalence of rotator cuff disease and the ongoing debate regarding the optimal injectable therapy, it is important to synthesise available evidence. This narrative review aims to compare corticosteroid and PRP injections in rotator cuff tendinopathy, summarising results from randomised controlled trials (RCTs), comparative studies, and systematic reviews, and highlighting current controversies, clinical implications, and future directions.

## Review

Methods

Study Design

This narrative review synthesised comparative evidence on corticosteroid versus PRP injections for rotator cuff tendinopathy.

Eligibility Criteria

We included RCTs, prospective comparative studies, and systematic reviews/meta-analyses enrolling adults with rotator cuff tendinopathy (including partial-thickness tears or calcific tendinitis) that compared corticosteroid with PRP/plasma rich in growth factors (PRGF). We excluded animal studies, case reports, editorials, and non-shoulder tendinopathies.

Information Sources and Search Strategy

A narrative review methodology was used to synthesise current evidence comparing corticosteroid and PRP injections for rotator cuff tendinopathy. A comprehensive search of Ovid MEDLINE, Embase, and PubMed was conducted using keywords and MeSH terms related to rotator cuff tendinopathy, PRP, and corticosteroids. The search was limited to human studies published in English from 2010 to 2025. The complete search strategy is available upon request.

The following search terms were used:

Population: "rotator cuff injuries", "tendinopathy", "supraspinatus", "infraspinatus"

Intervention: "platelet-rich plasma", "PRP"

Comparator: "corticosteroid", "steroid", "triamcinolone", "methylprednisolone", "dexamethasone"

The search string was built using Boolean operators (AND/OR), and filters were applied to include only human studies, English language, and publications between 2010 and 2025. Study types included were RCTs, observational studies, and systematic reviews.

Selection Process

Two reviewers independently screened titles/abstracts and evaluated full texts. Disagreements were resolved by consensus.

Data Items and Outcomes

We extracted study design, sample size, PRP protocol (leukocyte content, activation, dose/number of injections, delivery site), corticosteroid type/dose, follow-up duration, and outcomes (pain, function, range of motion (ROM), failure/retreatment). Primary outcomes were validated patient-reported measures (e.g., VAS, Disabilities of the Arm, Shoulder and Hand (DASH), American Shoulder and Elbow Surgeons (ASES), Constant-Murley).

Risk of Bias and Certainty

Given the narrative design, no formal tool was applied; we report limitations as described by the original authors and consider heterogeneity qualitatively.

Synthesis Methods

Findings were narratively synthesised by study type (systematic reviews, RCTs, prospective studies) and time horizon (short term <3 months; mid/long term ≥6 months).

See Appendix for complete search strategies across databases.

Results

**Figure 1:**
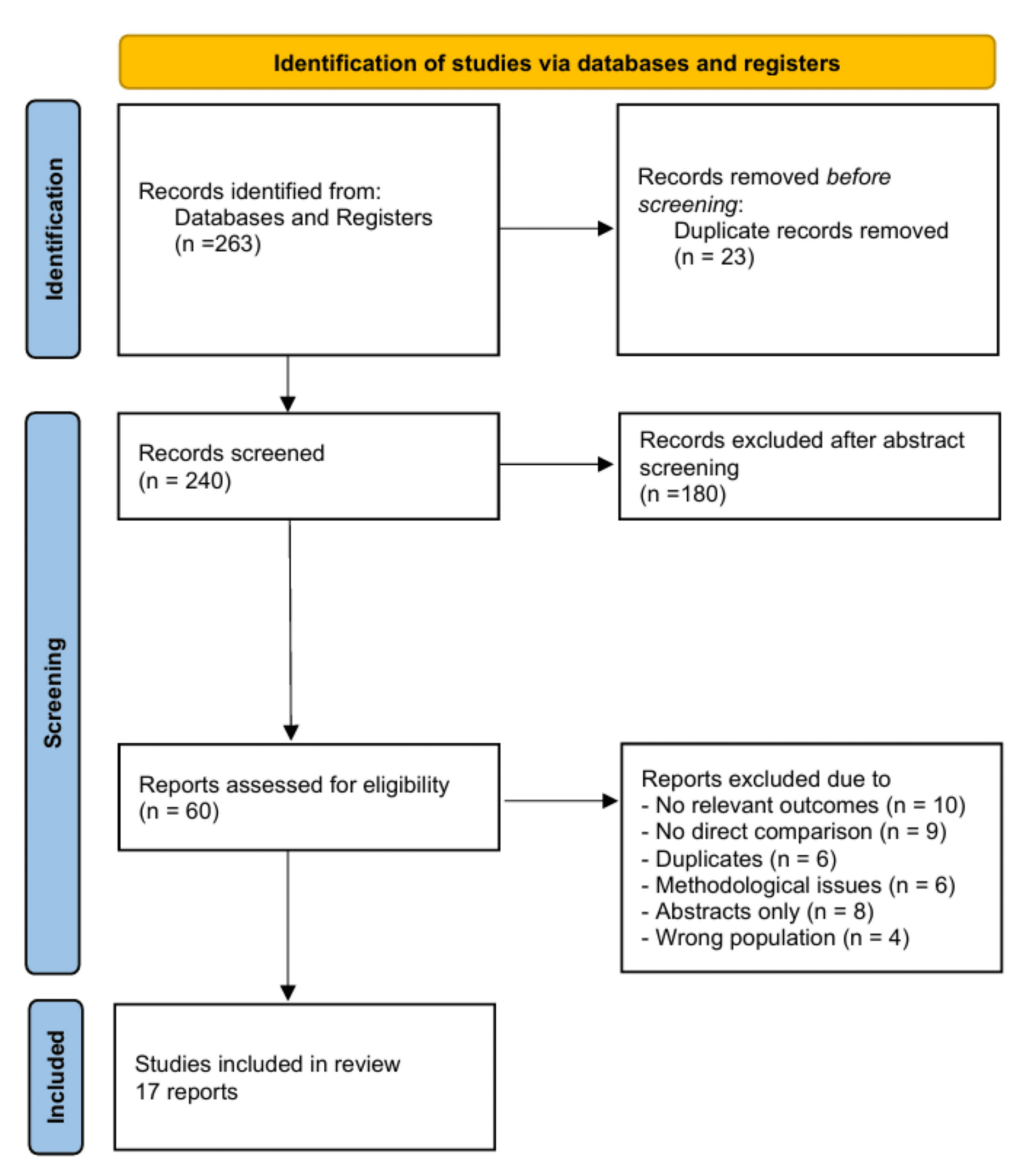
Study selection process The study selection process was based on the PRISMA 2020 flow diagram [17]. A total of 263 records was identified through database searches. After removal of duplicates and screening of titles and abstracts, 60 full-text articles were assessed for eligibility. Following application of inclusion and exclusion criteria, 17 studies were included in this narrative review. PRISMA: Preferred Reporting Items for Systematic Reviews and Meta-Analyses

The search initially identified 263 articles. After applying eligibility criteria, 60 full-text articles were assessed, of which 17 studies were included in this review (Figure 1). The included studies consisted of four systematic reviews/meta-analyses (Adra et al., Wang et al., Sun et al., Lin et al.), several RCTs (e.g., Kesikburun et al., Jo et al., Kwong et al., Rossi et al.), and prospective comparative studies (e.g., Annaniemi et al., Vaquerizo et al.) [3,5,7,9-11,13-16]. Data were narratively synthesised and grouped under systematic reviews, RCTs, and prospective studies to highlight short- and long-term clinical outcomes.

Systematic Reviews and Meta-Analyses

Several systematic reviews and meta-analyses have compared corticosteroid and PRP injections in rotator cuff tendinopathy. Additional meta-analyses similarly report that corticosteroids outperform PRP at short-term follow-up, whereas PRP may be favoured beyond 6 months, though results are inconsistent [8,12,18].

Adra et al. conducted a comprehensive meta-analysis of randomised trials and found that corticosteroids were favoured at short-term follow-up (<3 months), whereas PRP showed better outcomes at intermediate and medium-term follow-up (6-12 months) [5]. However, the differences did not reach clinical significance overall, and the authors highlighted heterogeneity in PRP preparation and delivery protocols as a limitation. Similarly, Wang et al. concluded that corticosteroids provided superior early pain relief, but there was no significant medium- or long-term difference between corticosteroid and PRP injections [7]. Sun et al., reviewing Chinese cohorts, reported no significant differences in pain, function, or ROM at short- or medium-term follow-up; however, at long-term follow-up, PRP demonstrated advantages over corticosteroids in reducing pain and improving function [13]. Finally, Lin et al. performed a network meta-analysis comparing multiple injectable therapies and found that corticosteroids were effective for short-term symptom relief (3-6 weeks), whereas PRP and prolotherapy tended to yield better long-term outcomes (>24 weeks) [3]. Given the heterogeneity across studies, the authors recommended cautious interpretation of these findings.

Overall, systematic reviews suggest that corticosteroid injections provide rapid but transient pain relief, while PRP and related biologics may offer more durable benefits, though results remain heterogeneous and not always clinically significant (Table 1). Network and pairwise meta-analyses comparing multiple injectables likewise rank PRP more favourably at longer follow-up [18].

**Table 1:** Systematic reviews/meta-analyses comparing PRP and corticosteroid injections in rotator cuff tendinopathy, with key findings and notes on heterogeneity. PRP: Platelet-rich plasma; ROM: Range of motion; HA: Hyaluronic acid

Study	Scope	Design	Key Findings	Notes
Adra et al. [5]	PRP vs corticosteroid in rotator cuff disease	Systematic review & meta-analysis	PRP favoured for mid- or long-term pain/function; corticosteroid better short-term; heterogeneity noted	Although corticosteroid favoured short term and PRP favoured mid-term, differences were not clinically significant overall.
Wang et al. [7]	PRP vs corticosteroid for conservative rotator cuff lesions	Systematic review & meta-analysis	Corticosteroid superior early; PRP superior at 6 & 12 months for pain and function	Corticosteroid effective early; no significant medium- or long-term difference.
Sun et al. [13]	PRP vs corticosteroid in rotator cuff tendinopathy	Meta-analysis	PRP shows superior long-term pain reduction vs corticosteroid	Chinese cohorts; heterogeneity persists. No difference short- or medium- term; no difference in ROM.
Lin et al. [3]	Multiple injectables for rotator cuff tendinopathy	Systematic review & network meta-analysis	Corticosteroid effective short-term; PRP ranks higher mid- and long-term among injectables	Includes HA and saline comparators. Heterogeneity; interpret with caution.

RCTs

Multiple RCTs have directly compared PRP with corticosteroids in rotator cuff tendinopathy. Earlier randomised trials in subacromial pain also compared PRP directly with corticosteroid [19,20] (Table 2). Kesikburun et al. found no significant differences between PRP and placebo at 1-year follow-up in chronic tendinopathy, leading to early scepticism about PRP efficacy [9]. Jo et al. reported that some functional scores (e.g., DASH, external rotation) improved more with allogeneic PRP than corticosteroids at 6 months, although PRP was not definitively superior overall [15]. Kwong et al. demonstrated that patients with partial-thickness tears or tendinopathy achieved superior pain relief and function with PRP at short-term follow-up (3 months), but the benefit was not sustained at 12 months [10]. Oudelaar et al. studied PRP as an adjuvant after needle aspiration of calcific deposits [21]. PRP was associated with worse outcomes at 6 weeks but better outcomes at 6 months. At 1 and 2 years, results were comparable between groups, with PRP reducing the need for additional treatment but being linked to more complications. Rossi et al. contributed a series of trials [11,22,23]. In refractory tendinopathy, PRP significantly reduced pain, improved function, and resolved sleep disturbances, with many athletes returning to sport [22]. In partial supraspinatus tears, outcomes were significantly worse compared with isolated tendinopathy [23]. Their most recent double-blind RCT found that PRP provided significantly greater improvements at 1 year, with a lower failure rate compared with corticosteroids [11]. Vaquerizo et al. studied PRGF, a derivative of PRP, and found no significant differences compared with corticosteroids, although PRGF was considered a safe alternative [14]. Smaller RCTs, including those by Ibrahim et al. and Dadgostar et al., reported broadly similar results between PRP and corticosteroids, with PRP showing some advantages in pain, ROM, and ultrasound findings [24,25].

Collectively, RCT evidence has shifted over the past decade. While early studies showed little benefit of PRP, more recent high-quality trials suggest PRP may offer superior outcomes in selected patients, particularly in the medium to long term.

Prospective Comparative Studies

Several prospective and observational studies add to the evidence base. Annaniemi et al. found that both PRP and corticosteroids improved symptoms, but there was no significant difference in outcomes at up to 18 months [16]. The authors concluded PRP may be a safe alternative. Kumar et al. and Saleem et al., in smaller prospective studies, reported more sustained improvements in pain, function, and ROM with PRP compared with corticosteroids [26,27]. Although limited by sample size and methodological variability, these studies broadly support the findings of RCTs that PRP may provide longer-lasting improvements in some patients.

Overall Trends

The collective evidence indicates a consistent pattern. Corticosteroid injections are effective for short-term pain relief but their benefits often diminish over time. PRP injections appear to provide more durable improvements in pain and function, particularly beyond 6 months. Response may vary depending on the type of rotator cuff pathology, with isolated tendinopathy responding more favourably than partial tears. Variability in PRP preparation, dosing, and injection technique remains a key limitation across the literature. Recent umbrella reviews across tendinopathies suggest mid-term advantages for PRP with uncertain long-term superiority [28].

**Table 2:** Randomised and prospective comparative studies of PRP versus corticosteroid injections in rotator cuff tendinopathy: design, population, follow-up, and key outcomes PRP: Platelet-rich plasma; RCT: Randomised controlled trial: PRGF: Plasma rich in growth factors; ROM: Range of motion

Study	Design	Population/Subtype	Follow-up	Key Findings
Kesikburun et al. [9]	RCT (PRP vs placebo)	Chronic rotator cuff tendinopathy	Up to one year	No significant difference vs placebo; early neutral PRP data (contextual).
Jo et al., 2020 [15]	RCT (allogeneic PRP vs corticosteroid)	Rotator cuff disease	Short- to mid-term	Some functional measures improved, but not definitively superior overall.
Kwong et al. [10]	Double-blind RCT (PRP vs corticosteroid)	Partial-thickness tears or tendinopathy	Short-term primary; extended follow-up	PRP superior for short-term pain/function vs corticosteroid; no sustained benefit at 12 months.
Oudelaar et al. [21]	Double-blind RCT (PRP adjunct after barbotage)	Calcific rotator cuff tendinitis	Two years	Early outcomes worse, similar by one to two years; more complications with PRP.
Ibrahim et al. [24]	Comparative (ultrasound-guided PRP vs corticosteroid)	Rotator cuff tendinopathy	Short- to mid-term	PRP is a good alternative to corticosteroid injection that promotes healing and decreases inflammation.
Rossi et al. [22]	Prospective/Comparative	Refractory rotator cuff tendinopathy	Mid-term	PRP decreased pain and improved function, resolved sleep issues; athletes returned to sport vs baseline/controls
Rossi et al. [23]	Comparative	Partial supraspinatus tear vs isolated tendinopathy	Mid-term	PRP less effective in partial tears than isolated tendinopathy.
Rossi et al. [11]	Double-blind RCT (PRP vs corticosteroid)	Rotator cuff tendinopathy	One year	PRP superior to corticosteroid at 1 year (pain and function); lower failure rate in PRP group.
Vaquerizo et al. [14]	Double-blind RCT (PRGF vs corticosteroid)	Chronic rotator cuff tendinopathy	One year	No significant difference; PRP safe alternative.
Annaniemi et al. [16]	Comparative (up to 18 months)	Rotator cuff tendinopathy	Up to 18 months	No significant difference.
Dadgostar et al. [25]	RCT (PRP vs corticosteroid)	Rotator cuff tendinopathy	Short- to mid-term	Similar overall; some advantage in pain/ROM.
Kumar et al. [26]	Comparative	Rotator cuff tendinopathy	Short- to mid-term	PRP superior in sustained measures.
Saleem et al. [27]	Comparative	Rotator cuff tendinopathy	Short- to mid-term	PRP favoured over corticosteroid.

Discussion

Study Characteristics

Across included studies, participants were predominantly adults over 40 years with symptomatic rotator cuff tendinopathy or partial-thickness tears after failed conservative care. Corticosteroid comparators most commonly involved subacromial triamcinolone or methylprednisolone delivered under image guidance, whereas PRP protocols varied in leukocyte content (leukocyte-rich (LR) vs leukocyte-poor (LP)), activation, dose/number of injections, and targeting (subacromial bursa vs intratendinous). Follow-up horizons clustered at 3-12 months (fewer studies extended to 24 months). Outcomes were primarily patient-reported (VAS, ASES, Constant-Murley, DASH), with some trials reporting retreatment/failure and imaging. This methodological and protocol heterogeneity-together with differences in pathology mix (isolated tendinopathy vs partial-thickness tear; calcific tendinitis)-likely underpins the inconsistent mid- to long-term signals across RCTs and cohorts (Table 2) [9-11,14-16,21-27].

Pathophysiological Rationale

Corticosteroids act via glucocorticoid-mediated suppression of inflammatory cascades, rapidly reducing pain and disability. However, basic and translational evidence indicates potential adverse effects on tendon biology with repeated or intratendinous exposure, including impaired collagen synthesis and tenocyte viability, which may translate clinically into diminishing returns over time [9,29,30]. PRP concentrates platelet-derived growth factors and cytokines that can modulate inflammation, stimulate fibroblast/tenocyte proliferation, promote angiogenesis, and support extracellular matrix remodelling mechanisms that align with the more durable improvements reported in several trials and reviews [10,22,31,32]. Critically, “PRP” is not a single product: leukocyte content, activation method, dose, number of injections, and target tissue (bursal vs intratendinous) vary across studies and likely influence clinical effect size and time course [10,11,14-16,22]. Pathology phenotype also matters. PRP responses appear stronger in isolated tendinopathy than in partial supraspinatus tears, and calcific tendinitis shows a biphasic pattern (transient early worsening followed by improvement), mirroring trial findings [21-23]. These mechanistic differences provide a coherent explanation for the time-dependent crossover seen in syntheses-corticosteroid superiority in the short term vs possible PRP durability by 6-12 months-and for discordant results when protocols and populations differ [3,5,7,10-13,18,22,23].

Corticosteroid Injections: Short-Term Efficacy and Safety

Systematic reviews and RCTs consistently demonstrate that subacromial corticosteroid injections provide rapid short-term relief (<3 months) in pain and function for rotator cuff tendinopathy [3,5,7,10-13,18]. This advantage typically attenuates beyond the early window, with several syntheses and trials showing convergence toward PRP or no between-group difference at later follow-up [3,5,7,10-13]. Clinically, a single image-guided subacromial injection can facilitate rehabilitation in acute flares; however, repeated injections are associated with diminishing benefit and potential tendon-related risks, supporting a cautious approach to serial dosing [9,29,30,33]. Short-term gains are robust on patient-reported outcome measures (PROMs) (Visual Analogue Scale (VAS), ASES, Constant-Murley, DASH), but consistent structural healing signals are not established. Guidelines continue to emphasise exercise-based care, with injectables viewed as adjuncts rather than stand-alone solutions [33,34].

PRP Injections: Protocol Considerations and Durability

Recent RCTs and comparative studies suggest PRP may yield more sustained improvements at 6-12 months in selected patients, though the effect is not universal [10-12,14-16,22,23,26,27]. Positive trials commonly employed ultrasound-guided subacromial injections; durability appears greater in isolated tendinopathy than in partial-thickness tears, where outcomes are attenuated [22,23]. In calcific tendinitis managed with barbotage, PRP has shown worse very-early outcomes but improved mid-term results, with groups appearing similar by 1-2 years [21]. Heterogeneity in leukocyte content, activation, dosing, and targeting likely contributes to inconsistent findings across studies and meta-analyses [3,5,7,10-16,18,22,23]. Standardised reporting of PRP characteristics and delivery is needed to clarify dose-response and optimise protocols.

Patient Selection and Image Guidance

**Figure 2:**
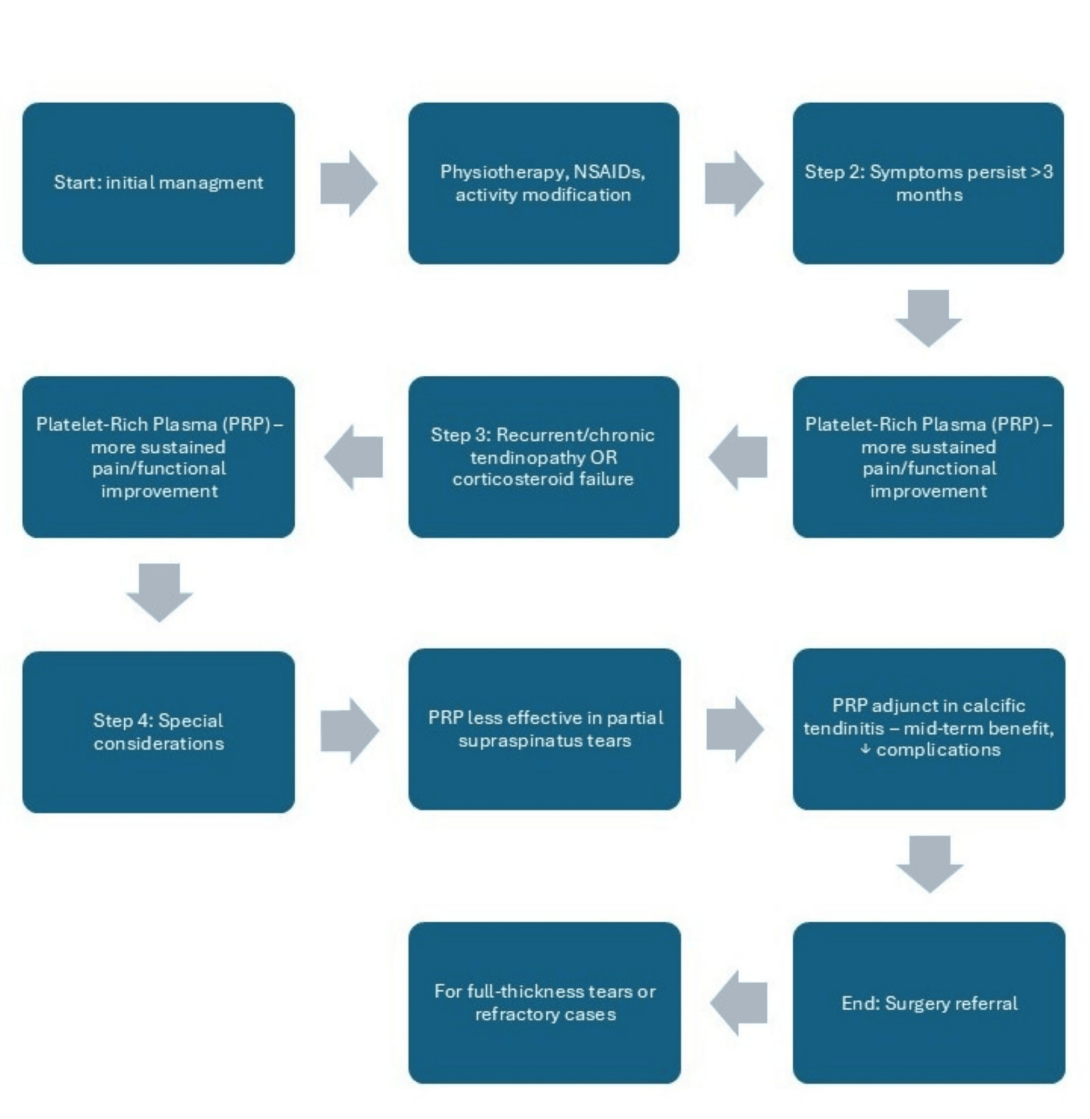
Proposed clinical decision algorithm for corticosteroid vs PRP injections in rotator cuff tendinopathy (based on current evidence) Image created by the authors. This figure is original and may be reused freely with appropriate citation. PRP: Plasma-rich platelet

Patients with chronic tendinopathy who prioritise durability and have no high-grade partial tears may be more likely to benefit from PRP, whereas those needing rapid analgesia (e.g., to start rehab) may prefer an initial corticosteroid injection [10-12,22,23,26,27,33,34]. Image guidance (ultrasound) is advisable for either modality to improve accuracy and document pathology (bursal inflammation, intratendinous disease, partial-thickness tear) [10-12,21-23]. A pragmatic, evidence-informed pathway is illustrated in Figure 2, aligning treatment choice with symptom time-course, pathology subtype, and patient preferences.

Adverse Events and Cost/Access

Serious adverse events are uncommon for both modalities. Corticosteroids can cause post-injection flare, skin changes, and-particularly with repeats-concerns about tendon health [9,29,30]. PRP is generally well tolerated but may produce transient pain flares or chemical bursitis; in calcific tendinitis trials, early frozen-shoulder events were reported more frequently in PRP groups before convergence at later time points [21,22]. From a health-system perspective, corticosteroids are inexpensive and widely available, whereas PRP often incurs out-of-pocket costs, which may limit access despite potential mid-term benefits [3,5,7,10-12,14-16,18,22,23,26,27,33-35]. High-quality cost-effectiveness data remain limited.

Limitations of the Current Literature

Several limitations must be acknowledged. There is substantial heterogeneity in PRP preparation, with differences in leukocyte content, activation methods, number of injections, and delivery sites (subacromial versus intratendinous). This variability complicates comparisons and may explain the neutral findings of some early studies such as Kesikburun et al. [9]. Many RCTs and prospective studies are small in scale, limiting statistical power. Most studies report outcomes only up to one year, with few long-term follow-ups beyond two years. Finally, economic evaluations are scarce, leaving the cost-effectiveness of PRP uncertain in different healthcare settings.

Future Directions

Future research should prioritise standardisation of PRP preparation, dosing and delivery techniques to enhance reproducibility and comparability. Large, multicentre RCTs with long-term follow-up are needed to clarify durability of outcomes and identify patient subgroups most likely to benefit. In parallel, cost-effectiveness analyses are required to evaluate the feasibility of PRP use in routine care. Future studies should report PROMs consistently, stratify by pathology type (isolated tendinopathy vs partial tears), and include health economic evaluations.

Study Limitations

This narrative review is limited by heterogeneity in PRP protocols (leukocyte content, activation, dose, and injection site), variable comparator steroid types/doses, small single-centre RCTs, and inconsistent follow-up horizons (few >12-24 months). The absence of a formal risk-of-bias assessment limits certainty in pooled interpretation. Cost-effectiveness data remain sparse.

## Conclusions

Corticosteroid and PRP injections represent two widely used interventions for rotator cuff tendinopathy, each with distinct strengths. Corticosteroids provide effective short-term relief but are limited by diminishing benefits and potential adverse effects with repeated administration. PRP may offer more durable improvements in pain and function, as suggested by several recent randomised trials and systematic reviews, though findings remain inconsistent and influenced by methodological variability. Variability in PRP preparation and delivery remains a key challenge, and current evidence is insufficient to establish standardised treatment protocols. Clinical decision-making should therefore be individualised, balancing the need for rapid symptom control with the potential for long-term tendon healing. Further high-quality, multicentre studies with standardised PRP protocols and extended follow-up are needed to clarify its role within treatment algorithms and determine whether it can complement or, in selected cases, replace corticosteroids.

## Appendices

A comprehensive search of Ovid MEDLINE, Embase, and PubMed was conducted using keywords and MeSH terms related to rotator cuff tendinopathy, PRP, and corticosteroids. The search was limited to human studies in English published from 2010 to 2025. RCTs, observational studies, and systematic reviews were included. The complete search strategy executed in August 2025 is as below.

**Table 3:** Search strategy

Database	Search Strategy
Ovid MEDLINE	1. exp Rotator cuff Injuries/ 2. (rotator cuff or supraspinatus or infraspinatus or tendinopathy or tendinitis or tendinosis).ti,ab. 3. 1 or 2 4. exp Platelet-Rich Plasma/ 5. (platelet rich plasma or PRP).ti,ab. 6. 4 or 5 7. exp Adrenal Cortex Hormones/ 8. (corticosteroid* or steroid* or triamcinolone or methylprednisolone or dexamethasone or hydrocortisone).ti,ab. 9. 7 or 8 10. 3 and 6 and 9 11. limit 10 to (humans and English language) 12. limit 11 to (randomized controlled trial or controlled clinical trial or systematic review or observational study or meta-analysis) 13. limit 12 to yr = "2010 – Current" 14. remove duplicates from 13
PubMed	(“Rotator Cuff Injuries”[Mesh] OR rotator cuff[tiab] OR supraspinatus[tiab] OR infraspinatus[tiab] OR tendinopathy[tiab] OR tendinitis[tiab]) AND (“Platelet-Rich Plasma”[Mesh] OR platelet rich plasma[tiab] OR PRP[tiab]) AND (“Adrenal Cortex Hormones”[Mesh] OR corticosteroid*[tiab] OR steroid*[tiab] OR triamcinolone[tiab] OR methylprednisolone[tiab] OR dexamethasone[tiab]) AND (“randomized controlled trial”[Publication Type] OR “controlled clinical trial”[Publication Type] OR “systematic review”[Publication Type] OR “observational study”[Publication Type] OR meta-analysis[pt] OR clinical trial[pt]) Filters: Humans; English; 2010–2025
Embase (via Ovid)	1. 'rotator cuff tendinitis'/exp OR rotator cuff:ti,ab OR supraspinatus:ti,ab OR tendinopathy:ti,ab 2. 'platelet rich plasma'/exp OR 'platelet rich plasma':ti,ab OR PRP:ti,ab 3. 'corticosteroid'/exp OR corticosteroid*:ti,ab OR triamcinolone:ti,ab OR methylprednisolone:ti,ab 4. 1 and 2 and 3 5. limit 4 to (humans and English language and yr = "2010 – Current") 6. limit 5 to (randomized controlled trial or controlled clinical trial or systematic review or observational study or meta-analysis) 7. remove duplicates from 6
